# Forest cover mapping in post-Soviet Central Asia using multi-resolution remote sensing imagery

**DOI:** 10.1038/s41598-017-01582-x

**Published:** 2017-05-02

**Authors:** He Yin, Asia Khamzina, Dirk Pflugmacher, Christopher Martius

**Affiliations:** 1 Center for Development Research (ZEF), University of Bonn, Walter-Flex-Str. 3, 53113 Bonn, Germany; 2 SILVIS Lab, Department of Forest and Wildlife Ecology, University of Wisconsin-Madison, 1630 Linden Drive, Madison, WI 53706 USA; 3 Division of Environmental Science and Ecological Engineering, Korea University, 145 Anam-Ro, Seongbuk-Gu, Seoul 02841 Korea; 4 Geography Department, Humboldt-Universität zu Berlin, Unter den Linden 6, 10099 Berlin, Germany; 5 Center for International Forestry Research (CIFOR), P.O. Box 0113 BOCBD, Bogor, 16000 Indonesia

## Abstract

Despite rapid advances and large-scale initiatives in forest mapping, reliable cross-border information about the status of forest resources in Central Asian countries is lacking. We produced consistent Central Asia forest cover (CAFC) maps based on a cost-efficient approach using multi-resolution satellite imagery from Landsat and MODIS during 2009–2011. The spectral-temporal metrics derived from 2009–2011 Landsat imagery (overall accuracy of 0.83) was used to predict sub-pixel forest cover on the MODIS scale for 2010. Accuracy assessment confirmed the validity of MODIS-based forest cover map with a normalized root-mean-square error of 0.63. A general paucity of forest resources in post-Soviet Central Asia was indicated, with 1.24% of the region covered by forest. In comparison to the CAFC map, a regional map derived from MODIS Vegetation Continuous Fields tended to underestimate forest cover, while the Global Forest Change product matched well. The Global Forest Resources Assessments, based on individual country reports, overestimated forest cover by 1.5 to 147 times, particularly in the more arid countries of Turkmenistan and Uzbekistan. Multi-resolution imagery contributes to regionalized assessment of forest cover in the world’s drylands while developed CAFC maps (available at https://data.zef.de/) aim to facilitate decisions on biodiversity conservation and reforestation programs in Central Asia.

## Introduction

Central Asia comprises five predominantly agricultural countries of the former Soviet Union (Kazakhstan, Kyrgyzstan, Tajikistan, Turkmenistan, and Uzbekistan) that, although largely covered by drylands, are home to diverse and important forest ecosystems, ranging from continuous forests in the higher-rainfall upstream areas to patchy riparian forests in the more arid downstream areas^1–3^. Notwithstanding their relatively small coverage area, forests in Central Asia play a key role in the regional hydrology by helping maintain a steady river discharge from the high mountains to the irrigated lowlands^4^. Mountainous forests are particularly rich in terms of the genetic diversity of wild fruit and nut tree species and serve as carbon sinks^5–8^. Given their great ecological and environmental importance, most of the forests in Central Asia have been placed in Group I of the Soviet Union forestry system, which is designated for conservation and protection^4, 9–12^. However, following the collapse of the Soviet Union, regular and standardized forest inventories have stopped, with the effect that the status and condition of these forests is currently uncertain.

Forest statistics provided by these Central Asian countries to the Food and Agriculture Organization of the United Nations (FAO) are mostly out-dated or missing. For example, the country report by Uzbekistan states that the last forest inventory was conducted in 1987; no national statistics are provided by Turkmenistan; Kazakhstan reports results based on a desk study (http://www.fao.org/countryprofiles/en/); and in general, the capacities of these countries for forest monitoring is unknown^13–15^. There are substantial discrepancies between datasets generated from different sources, with the effect that there is no commonly agreed upon estimate for the total forested area in Central Asia^4^. Based on individual country reports, the Global Forest Resources Assessments (FRA)^16^ estimates that 11.7 million ha, or less than 5% of the land area, are covered by forest in Central Asia. However, official statistics often overestimate the total forested area and do not reflect the actual forest condition^17^. The use of different definitions for forest, some of which deviate from international definitions, introduces further ambiguities between estimates from Central Asian countries^18–20^. Spatially explicit, comparable methodologies for obtaining forest information are much needed in Central Asian countries, so that high quality datasets can be established that will enable adequate assessment of ecosystem services (e.g., biodiversity, climate, and water cycle regulation) and development of sustainable management options^21^.

Remote sensing provides seamless and periodic observations of the Earth, and for countries with few or no inventories, it is the only viable source of forest area estimates e.g.^22^. The independence of remote sensing from national forest agencies is an additional advantage for forest monitoring across political boundaries^23^. Several continental and global land cover mapping efforts based on coarse resolution satellite data (i.e., spatial resolutions greater than 250 m) provide a number of land cover products, such as the GLC2000^24^, GlobCover^25^, and MODIS Land Cover Type^26, 27^ products. These datasets map the distribution of forest types with acceptable accuracies on a global scale but, due to their coarse resolution, are insufficient for national forest area reporting^28^. Products that estimate the sub-pixel proportion of forest area, such as MODIS Vegetation Continuous Fields (VCF)^29^, seek to overcome the coarse resolution bias^30, 31^. However, the accuracy of global maps can vary substantially between regions^32–39^. It is difficult and not always feasible to capture regionally important ecological gradients with global products that are based on globally selected reference data^26, 40, 41^. Large discrepancies between global land cover products have been found, especially at biome borders and in sparsely vegetated environments^42, 43^.

With recent developments in data access and distribution policies for Landsat and Sentinel-2, improved forest monitoring using medium-resolution satellite image time series data has become feasible^44, 45^. Medium-resolution sensors capture more spatially detailed information, which is important for forest monitoring in heterogeneous drylands^46, 47^. In addition, using multi-temporal instead of single-date imagery allows for more accurate land cover classification^48–50^. Recently, the concomitant use of all available Landsat imagery time series data has provided opportunities for large-area forest cover mapping^23, 51, 52^. Taking advantage of all available observations per pixel, it is possible to use Landsat time series data for improved classification^53–55^.

Combining medium- and coarse-resolution imagery can support cost-effective forest mapping over large areas^56–58^. In spite of the rapid developments in computational capability, processing the large volumes of data associated with higher resolution images is still challenging. In addition, data scarcity due to low observation density and persistent cloud and snow coverage make it difficult to use Landsat to produce wall-to-wall forest cover maps^59, 60^. Therefore, coarse resolution archive imagery, such as from AVHRR and MODIS, has been combined with Landsat data for the land cover mapping of large areas^56, 57, 61–63^. The higher resolution of Landsat makes it feasible to estimate the percentage of different land cover types at the MODIS level of coverage and predict wall-to-wall sub-pixel forest cover through regression or other approaches^64–67^.

The goal of this study was to develop a consistent and improved forest cover map for Central Asia, and to assess the current national and global forest cover estimates for this region. To achieve this, we first produced land cover maps from a sample of Landsat footprints corresponding to the World Reference System 2. We then used the Landsat-based map as a reference to train a fractional forest cover model based on MODIS time series data at a resolution of 250 m. The objectives of this study were thus to: 1) assess the usefulness of dense Landsat time series data for forest cover mapping in drylands; 2) produce a Central Asia Forest Cover (CAFC) map using MODIS time series data for 2010 that distinguishes the prevalent forest types; and 3) compare this regionalized forest cover map with the FRA and other available global coverage products to evaluate the relative strengths and limits of all available tools for forest monitoring in Central Asia.

## Results

### Classification of Landsat imagery

The use of spectral-temporal metrics generated from three years of imagery yielded the highest classification accuracy (overall accuracy OA: OA = 0.83 ± 0.04) while using single-date composite produced the lowest (OA: 0.56 ± 0.04) (see Supplementary Table S1, and Fig. S1). Using temporal metrics improved the separation of deciduous forest and herbaceous vegetation (see Supplementary Fig. S2). In addition, the confusion between mixed and evergreen forest decreased, resulting in improved mapping accuracy for these two classes. Extending the observation period from one to three years reduced the classification uncertainty further. Most of the land cover classes, particularly forest, exhibited fewer classification errors. Fewer cases of confusion occurred between forest and wetland (see Supplementary Fig. S2). Adding observations from 2009 and 2011 to the 2010 dataset led to more accurate distinctions between different forest classes, especially for deciduous forest. The data scarcity effect, e.g. the strip artefact of ETM + Scan Line Corrector (SLC)-off, was largely eliminated when including temporal metrics and the extended observation period (see Supplementary Fig. S2).

### Forest cover percentage prediction using MODIS

**Figure 1:**
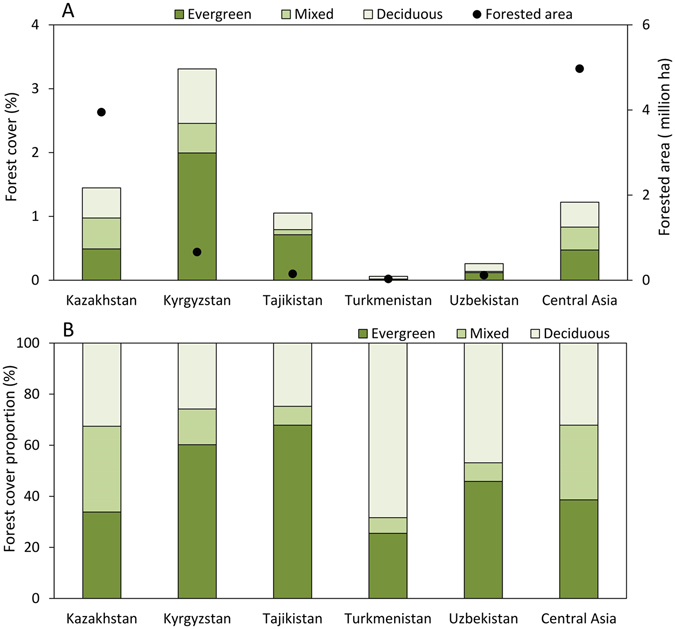
Land area covered by forests (**A**) and the composition of forest types (**B**) in Central Asia.

**Figure 2:**
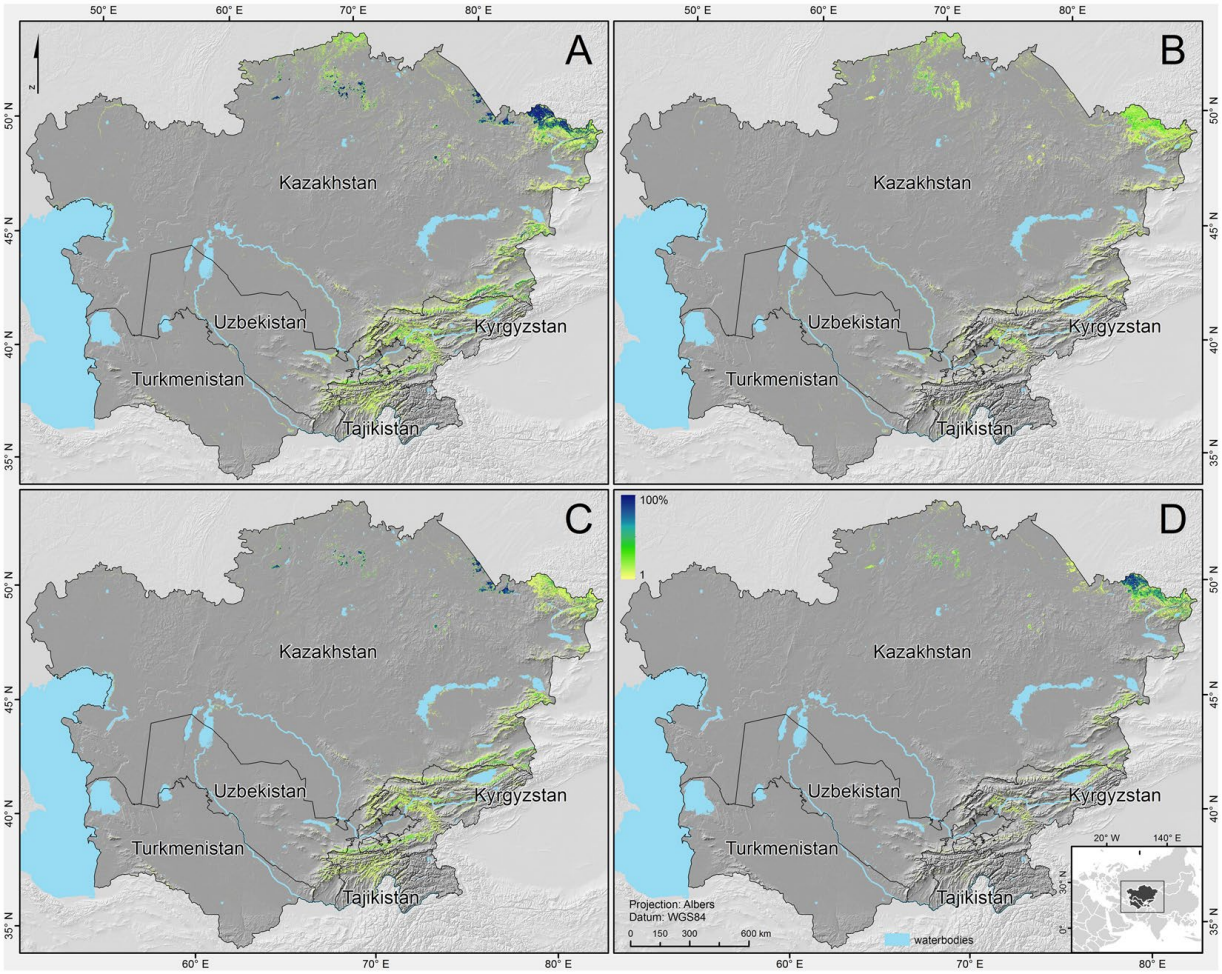
Forest cover percentage with (**A**) all forest types, (**B**) deciduous, (**C**) evergreen, and (**D**) mixed forest. This figure was produced using ArcGIS 10.3.

**Figure 3:**
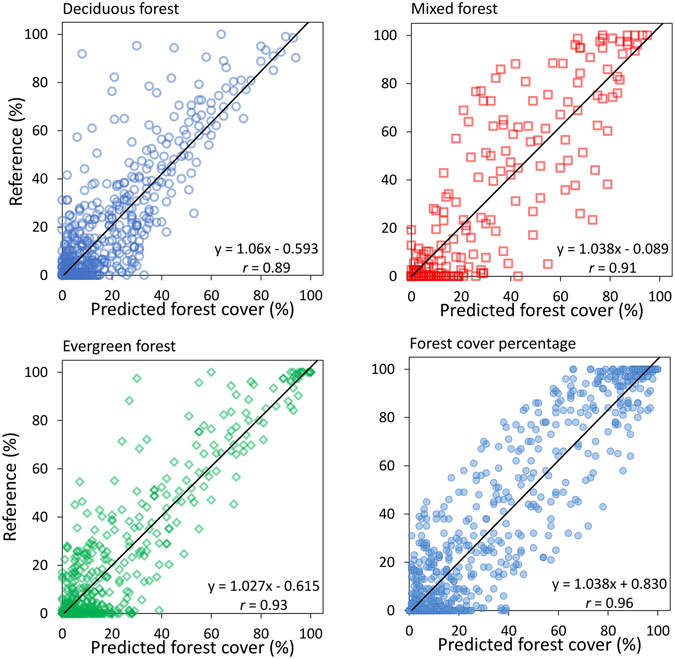
Scatterplots of reference forest cover derived from Landsat land cover maps and the estimated forest cover from MODIS data as deciduous, mixed, evergreen, and overall forest.

The Central Asia Forest Cover (CAFC) map produced from the MODIS data indicates that forest covers 4.96 million ha (1.24% of the land area in Central Asia) with a distinct variation in forest types between the countries (Figs 1 and 2). Kyrgyzstan was found to have the largest proportion of forest cover (3.3% of its land area), while Turkmenistan carried the least (0.06% of the land area). Due to its large territory, Kazakhstan is home to most of the forest (79.4% of the total forest in Central Asia), with forests relatively evenly distributed between deciduous, evergreen, and mixed forest types. In the mountainous countries Tajikistan and Kyrgyzstan, evergreen forests were more prominent, occupying 67.9% and 60.2% of the total forest area, respectively. Deciduous forests prevailed in Turkmenistan (68.3% of the country’s forest area), a much more arid country. Prediction accuracy varied by forest type (Fig. 3). Overall forest cover was predicted with the highest accuracy. The normalized root-mean-square error (NRMSE) of the predicted evergreen forest cover (NRMSE = 1.19) was lower than the errors for deciduous (1.35) and mixed forests (1.84). The overall forest cover map had the lowest NRMSE (0.63). The linear relationship between predicted forest cover and reference forest cover was stronger (Pearson’s *r* = 0.96) than that between the cover estimates of any single forest type and the reference.

### Comparison of forest cover estimates

**Figure 4:**
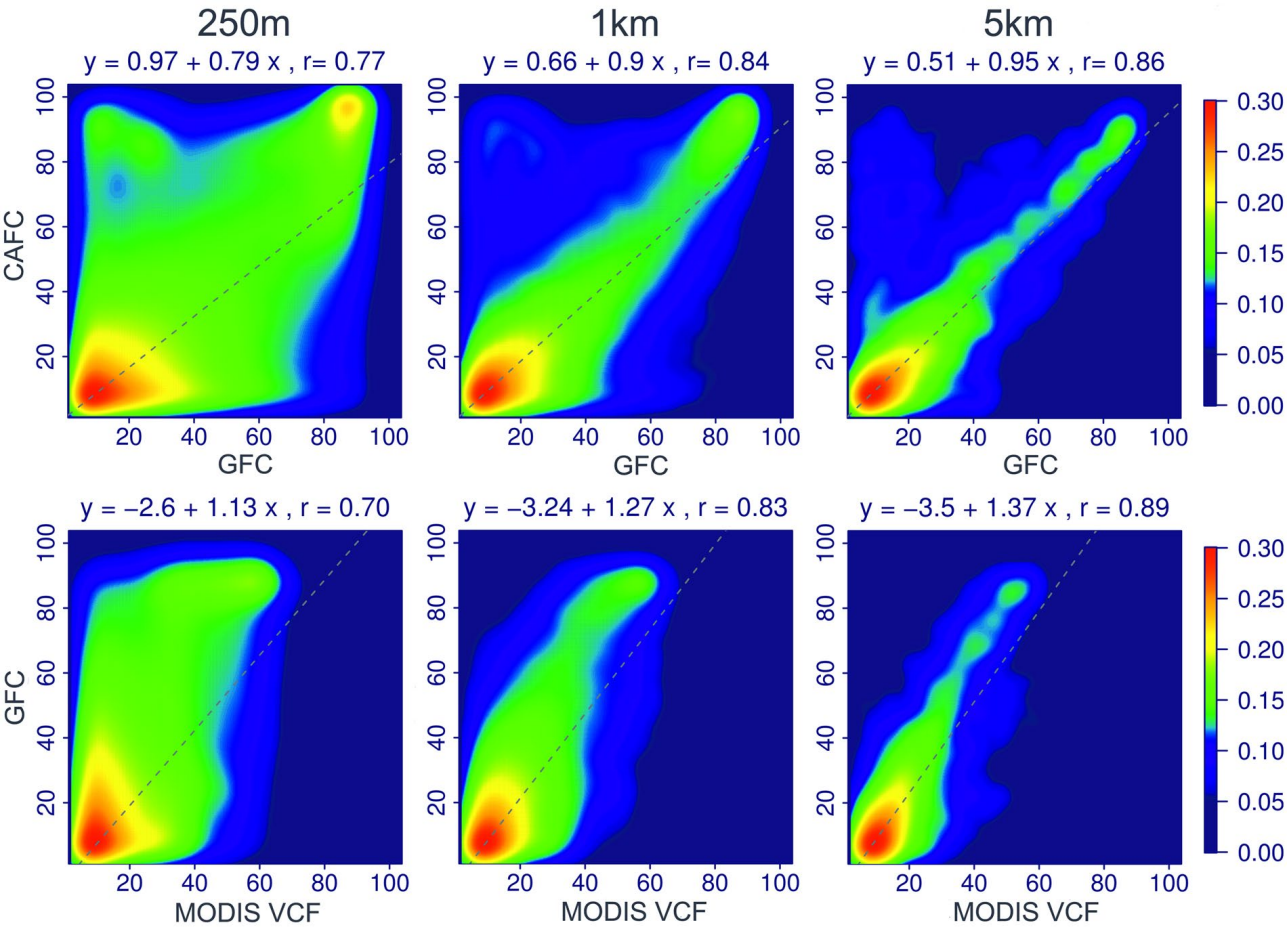
Joint distribution of forest cover percentage from CAFC vs. GFC (upper row) and tree cover percentage from MODIS VCF vs. GFC (lower row) at the sampling resolutions of 250 m, 1 km, and 5 km, with the colour scheme representing local densities at each point of the scatterplot (obtained through a kernel density estimate) and the dotted regression line demonstrating the coherence between different products.

Comparison of the forest cover estimates for Central Asia showed both agreement and discrepancies between the CAFC and the global products. In general, the CAFC produced patterns similar to GFC (Fig. 4; also see Supplementary Fig. S3). Although the GFC is more detailed because of its higher spatial resolution, we found localized but substantial over- and underestimations in this map. For example, GFC overestimated forest cover in non-woody wetlands (Figure S3, Ili River Delta) and tended to underestimate forest cover in riparian forests (Figure S3, Amu Darya) and evergreen forests (Figure S3, Eastern Kazakhstan Region). When comparing GFC and MODIS VCF, we found that MODIS VCF overestimated forest cover in regions with low forest cover and underestimated the dense forest areas in most of Central Asia (Fig. 4, see also Supplementary Fig. S3). Overall, the strong agreement between the CAFC and GFC further improved when we aggregated the maps to coarser spatial resolutions (Fig. 4). The correlation (Pearson’s *r*) between the CAFC and GFC increased from 0.77 at a pixel size of 250 × 250 m to 0.86 at 5 × 5 km. At a pixel size of 5 × 5 km, the regression slope was close to 1 (0.95). MODIS VCF and GFC had a strong positive relationship at pixel sizes of 250 × 250 m (*r* = 0.7) to 5 × 5 km (*r* = 0.89); however, MODIS VCF made lower tree cover predictions with regression slopes ranging between 1.13 and 1.37. Forest cover estimates from the CAFC and GFC tended to agree closely, with the exception of mountainous Tajikistan. MODIS VCF typically provided a much lower estimate, totalling 0.3% of Central Asia as a whole (Table 1). The FAO FRA 2010^68^ and FRA 2015^16^, relying on individual country reports, largely overestimated forest cover, with the exception of Kazakhstan and Kyrgyzstan in most recent assessment. For the other countries, the FRA estimates were between 1.5 and 147 times higher than the CAFC estimates, with particular extremes for the more arid, downstream countries of Turkmenistan and Uzbekistan.

**Table 1:** Forest cover percentage (%) in Central Asian countries estimated from the Central Asia Forest Cover map (CAFC), Global Forest Change (GFC), MODIS VCF, and Forest Resources Assessments (FRA).

Data source	Kazakhstan	Kyrgyzstan	Tajikistan	Turkmenistan	Uzbekistan
CAFC	1.45	3.3	1.05	0.06	0.29
GFC	1.59	3.42	0.43	0.02	0.24
MODIS VCF	0.41	0.54	0.02	0.00	0.01
FRA (2010)	1.2	5	2.9	8.8	7.7
FRA (2015)	1.2	3.3	3.0	8.8	7.3

## Discussion

Using all available Landsat imagery rather than single-date spectral reflectance, we were able to adequately distinguish between forests and other land cover classes with an overall accuracy of 0.83 ± 0.04, increased from 0.56 ± 0.04 when only one date was used. Metrics derived from time series data provide information on phenology patterns and therefore are less influenced by inter-annual variability in the imaging dates and overall image availability^55^. This is critical for land cover mapping in drylands characterized by high diversity in plant phenology. Moreover, metrics calculated from full time series are more resistant to the noise from geometric errors in single images^69^.

Extending the observation period from one year to three years (called epoch imagery) for land cover classification with the Landsat data further reduced mapping errors (OA: 0.83 ± 0.04 compared to 0.71 ± 0.04). Using MODIS time series data, Hüttich *et al*.^70^ arrived at similar accuracy levels with increasing length of observation period in the drylands of Africa. In contrast to the frequent MODIS observations, Landsat imagery has lower temporal resolution and the time series data has many gaps^45, 71^. Our study suggests that data scarcity due to sensor errors or frequent cloud/snow cover could be reduced by including imagery from neighbouring years. Furthermore, classification errors due to considerable intra-annual variations in dryland vegetation can be largely avoided by including multi-annual time series data from remote sensing observations^72^.

Extreme land cover conversions could produce large variations in a time series, thus undermining its suitability for use in epoch imagery for classification. To the best of our knowledge, there were no dramatic changes for the examined land cover categories in Central Asia during the observation period of 2009–2011. The mapping confusion between deciduous forest and herbaceous land was likely due to general landscape heterogeneity in Central Asia. Mixed land cover produces mixed pixels which are prone to classification errors^73^. Hindsight examination on the confused validation samples revealed that 53% of all deciduous forest/herbaceous land misclassifications were mixed pixels dominated by herbaceous vegetation and scattered trees. Another challenge is the separation of land cover classes with similar spectral and temporal responses. Mixed forest has a wide range of spectral reflectance and is thus often confused with deciduous or evergreen forests^74^. These uncertainties are a problem inherent to land cover mapping based on remote sensing in heterogeneous landscapes and can be largely reduced through the use of temporal metrics from multi-annual Landsat time series data. Compared to other forest cover maps our mapping approach using Landsat time series captures forest cover in drylands reasonably well (Supplementary Table S1). For example, the National Land Cover Database (NLCD) of the Conterminous United States, also using Landsat data, reported the forest user’s accuracy of 47% and the producer’s accuracy of 99% in the dryland region, covering the great plains and deserts^75^.

We found strong agreement between MODIS- and Landsat-based forest cover estimates indicating that MODIS can provide reliable data for forest cover monitoring in dryland forests. Although uncertainty was relatively high at the pixel level (e.g., an NRMSE of 0.63 for overall forest cover), the precision of the estimates improved when aggregated over larger areas. Using MODIS to estimate the proportional cover of different forest types in Central Asia revealed uncertainty due to contamination of the Landsat training dataset. Mapping errors also originated from training data imperfections^76^. Using a land cover map derived from high resolution imagery as a reference in the estimation of fractional land cover at coarse resolutions could lead to inter-resolution error propagation^46^. The slight overestimation of forest cover in the wetlands of the Ili River delta (see Supplementary Fig. S3) could be traced back to the residual error in the Landsat map (see Supplementary Fig. S2). The higher mapping error of deciduous forest in the Landsat map may decrease a mapping accuracy of the CAFC deciduous forest map (see Supplementary Fig. S1). The challenge of distinguishing between spectrally similar classes persisted at the MODIS scale, as shown by the relatively moderate mapping accuracy for mixed forest (Fig. 3). In the same vein, mapping accuracy was highest for the overall forest fractional cover, eliminating confusion errors between forest types (Fig. 3).

We detected considerable differences between the forest maps generated from continuous tree cover products for Central Asia. Saturation of spectral reflectance, phenological variation, and confusion with dense herbaceous vegetation are possible reasons for commission errors in sparsely covered areas and omission errors in densely covered regions in the MODIS VCF product^77, 78^. In contrast, GFC generally provided a reliable forest estimate in Central Asia. However, calibration, especially to correct mapping errors in riparian and evergreen forests, would be needed for regional applications of the GFC product in Central Asia. In our study model-based inferential framework was used to obtain forest area estimates^79, 80^. In contrast to design-based inference, the validity of the model-based inference was supported by the strong linear relationship (r = 0.96) and the coefficient close to 1. Both suggest the unbiasedness of our model-based area estimate. To overcome uncertainty in the map data, there is a need to integrate various resources such as ground-based measurements and satellite-derived maps to generate systematic forest cover estimates at a national level^28, 81^.

In general, the differences between the FRA estimates and all satellite-derived data were substantial. The greater forest cover reported by Turkmenistan and Uzbekistan in the FRA is at least partially attributed to these countries reporting sparse desert woodland as part of the national forest areas^18, 82^. Desert woodlands are characterized by low primary production, but are highly valuable in the regional ecology^18^, which explains the high importance given to them in the national forest reports.

The minimum area used in the forest definition could be another factor causing differences between the remote sensing estimates and the country reports. Applying a minimum mapping unit can influence forest area estimates from map data^20^, particularly in heterogeneous and fragmented landscapes. Forest inventories In Central Asian countries traditionally have not followed a fixed minimum area criterion hence various values were used, which might be above or below the uniform FAO requirement of >0.5 ha^16, 82^. In this study, we did not apply a minimum area requirement for the definition of a forest. Because the majority of the forests in Central Asia are contiguous areas, the effect is likely negligible or could even increase the difference between the remote sensing estimates and the country reports.

While countries might choose their own forest definition when reporting to the FRA, the large discrepancy in forest area estimates between the remote sensing assessment and the national statistics^68^ calls for a more coherent approach to forest monitoring across Central Asia. To this end, our proposed cost-effective approach using time series data from multi-resolution imagery and coherent maps of forest cover with 250 m resolution across Central Asia can complement and facilitate national forest inventories still largely dependent on resource demanding field surveys.

In particular, land cover classification using the Landsat imagery revealed the value of dense time series for land cover mapping in the drylands. With the release of more advanced medium resolution imagery such as the upcoming Sentinel-2 archive, uncertainty due to data scarcity might be further reduced. In contrast to the available global coverage products, the CAFC map provides information on prevalent forest types while considering the specific environmental settings of Central Asia. It could serve as a useful basis for standardized forest monitoring across country borders in Central Asia and, for example, carbon reporting to the United Nations Framework Convention on Climate Change (UNFCCC) and Reducing Emissions from Deforestation and Forest Degradation (REDD+). Moreover, our approach to the forest cover map for 2010 might be applicable to historic imagery, thus aiding in the continuous monitoring of forest cover changes in the Central Asian drylands.

## Methods

### Study area

Landlocked deep inside the Eurasian continent, Central Asia is dominated by a distinctive continental arid to semi-humid climate^83–85^. The sparsely vegetated deserts (Karakum and Kyzylkum) and the Eurasian Steppe belt stretching from the Caspian Sea to the Tian Shan Mountains cover most of the region, but a strong gradient in temperature and precipitation exists from north to south and from the lowlands to the mountains (see Supplementary Fig. S4). Associated with the diverse climates and terrains, forests in Central Asia consist of various types, with a rich species composition^3^. Dense needle-leaved evergreen forests are found in the mountains of Kazakhstan, Kyrgyzstan, and Tajikistan. The prevalent tree species include Scots pine (*Pinus sylvestris*), Siberian fir (*Abies sibirica*), Asian spruce (*Picea schrenkiana*), and junipers (*Juniperus* spp.). Broadleaved trees such as Tianshan birch (*Betula tianschanica*) and English oak (*Quercus robur*), and wild fruit and nut bearing species (e.g., walnut [*Juglans regia*], pistachio [*Pistacia vera*], and wild apple [*Malus sieversii*]) are more abundant in the lowlands and the lower mountain zone. Along the large river systems of the Amu Darya and Syr Darya, riparian forests dominated by Euphrates poplar (*Populus euphratica*) provide important corridors for wildlife and harbor a rich biodiversity^86^. Sparse stands of saxaul trees (*Haloxylon* spp.) and various xeric shrubs form “desert forests” in the sandy deserts of Turkmenistan and Uzbekistan^18^.

### Land cover classification from Landsat imagery

We randomly selected sixteen Landsat footprints covering the principal forest biomes of Central Asia based on the terrestrial ecoregions used by the World Wildlife Fund (WWF)^87^. Two extra footprints (152/31 and 154/31) for which ground data were available were added manually. We downloaded all available precision terrain corrected Landsat TM and ETM + L1T products with less than 100% cloud coverage for the period 2009–2011 for the eighteen Landsat footprints from the U.S. Geological Survey (USGS) (see Supplementary Fig. S4). Detailed information about the Landsat imagery used in this study can be found in Supplementary Table S2.

For each Landsat image, we performed atmospheric correction and radiometric calibration using the Landsat Ecosystem Disturbance Adaptive Processing System (LEDAPS) to ensure consistency between different sensors, dates, and footprints^88^. We used an object-based algorithm FMASK to generate cloud/shadow/snow masks for each image^89^. All imagery was then reprojected to a regional Albers equal-area conic projection.

To develop cloud- and gap-free forest cover maps with Landsat data for 2010, we built temporal composites using all available imagery. We tested three different composite types yielding three different sets of predictors (see Supplementary Table S3). The first set of predictors was based on a single date compositing strategy. For each reflectance band, a target day of the year (DOY) in mid-summer (DOY 210) was set and a clear observation closest to the target day was selected for compositing a gap-free clear image. The second set was comprised of all clear observations from 2010 and was used to calculate five metrics for each reflectance band: the mean, median, standard deviation, 25^th^ percentile, and 75^th^ percentile. The third set used the same statistical metrics as the second set, but for an expanded observation period of 2009 to 2011.

To map the forest composition in Central Asia, we classified forest types into deciduous forest, evergreen forest, and mixed forest (in which deciduous or evergreen tree species have a canopy cover of less than 60%). In addition to forests, we also mapped four other land cover classes, including non-woody wetland, herbaceous land (including grassland and cropland), barren land, and water bodies, to gain a deeper understanding of error sources when mapping forests in the drylands. The definition of forest and other land cover types was adapted from the IGBP DISCover dataset^90^. Because tree stands are relatively sparse in the drylands, we defined forest as lands dominated by tree cover greater than 30%.

We used the random forest (RF) classification algorithm^91^ to predict land cover for each set of predictors. The number of variables randomly sampled as candidates at each split was set to the square root of the number of input variables, and the minimum sizes of the terminal nodes and the number of the trees were set to 10 and 1000, respectively. Per-pixel class was predicted based on the majority of tree votes for a given class. The package ‘randomForest’^92^ implemented in the statistical software CRAN R^93^ was employed to conduct the analysis.

The training and validation samples for Landsat land cover mapping were selected independently from references collected in the field, high-resolution images from Google Earth and Advanced Spaceborne Thermal Emission and Reflection Radiometer (ASTER), for assessing Landsat imagery and MODIS time series acquired for the period 2009–2011. First, 4389 training polygons were labelled with the aid of high resolution imagery from Google Earth. To guarantee geometric accuracy, we cross-checked the geolocation of the polygons on ASTER imagery to ensure there were no major terrain displacements. Multi-date imagery, such as winter acquisitions together with the spring leaf-onset and autumn leaf-shading imagery (either from Google Earth, ASTER or Landsat), were used to better distinguish deciduous, evergreen, and mixed forests. Second, Landsat pixels within the training polygons were selected as the final training samples. In all, we created 24,238 training pixel samples, with 8,661 labelled as forest.

To avoid smaller samples for smaller classes such as forest or wetland, we used disproportionate stratified sampling at the Landsat pixel level for accuracy assessment^94^. First, we randomly selected 300 Landsat pixels as reference samples within each land cover class from the map produced from the temporal metric 2009–2011. Second, each sample was manually labelled by an expert interpreter, without knowledge of the mapped class label, using high resolution imagery from Google Earth, ASTER, and Landsat images acquired around 2010. In addition to the Landsat images, the interpreter also used temporal profiles of the MODIS NDVI time series (MOD13Q1) to aid in the labelling of samples with data gaps in the Google Earth, ASTER, and Landsat images. For each set of classifications, we constructed an error matrix from which we calculated overall accuracy, commission and omission errors. Finally, we selected the map with the best classification accuracy for use as a reference when predicting forest cover percentage using the MODIS time series.

### MODIS preprocessing and fractional forest cover mapping

Two MODIS Vegetation Index (VI) Collection 5 products from Terra (MOD13Q1) and Aqua (MYD13Q1) were used as the main sources for the production of the fractional forest cover map for Central Asia. The two products have a nominal spatial resolution of 250 m and contain 16-day composites of two vegetation indices and four spectral reflectance bands: the Normalized Difference Vegetation Index (NDVI), Enhanced Vegetation Index (EVI), surface reflectance in the blue, red, near-infrared (NIR), and shortwave-infrared (SWIR) wavelengths. We downloaded all imagery for eight MODIS tiles (h21v03, h22v03, h23v03, h21v04, h22v04, h23v04, h22v05, and h23v05) covering the entire Central Asia land area (see Supplementary Fig. S4) between January and December 2010 from the United States Geological Survey (USGS) Land Processes Distributed Active Archive Center (LPDAAC, available: https://mrtweb.cr.usgs.gov/). All imagery was reprojected to an Albers equal-area conic projection.

To increase the observation density, we combined the indices and spectral reflectance acquired from Terra and Aqua to achieve a temporal resolution of 8 days. The combined time series was reconstructed based on the order of the DOY information provided along with the MOD13Q1/MYD13Q1 spectral reflectance. To reduce residual noise in the time series caused by clouds, ozone, dust, off-nadir viewing, and low sun zenith angles, we smoothed the data using a Savitzky-Golay filter in the TIMESAT software^95^. Pixels flagged as no data, snow/ice, or cloud in the MOD13Q1/MYD13Q1 pixel reliability layer were excluded prior to the filtering. Outliers that were not identified as such in the MODIS reliability layer were removed with a seasonal-trend decomposition based on loess (STL)^96^, which is global in character and not dependent on ancillary data. We calculated several seasonal statistics from the MODIS time series to serve as predictor variables. For each smoothed MODIS time series (NDVI, EVI, and the four reflectance bands), we computed the mean, minimum, maximum, range, and standard deviation for spring (March-May), summer (June-August), and autumn (September-November).

The Landsat land cover map served as a source for the generation of training data. First, we randomly selected MODIS pixel samples, during which we enforced a minimum distance of 1000 m between samples to avoid spatial autocorrelation and along-scan triangular point spread function (PSF) effects^97^. Second, we calculated the proportion of each forest type in each sample using the Landsat land cover map. As a result, we obtained 16,173 training samples comprising the percentage cover of each forest type to train the MODIS forest cover model.

We applied Random Forest (RF) regression using the R-package randomForest^92^ implemented in R^93^ to predict per-pixel forest cover percentage. While RF classification estimates class membership of categorical data by applying a majority vote to individual tree predictions, RF regression predicts a continuous response by averaging individual tree predictions per-pixel. The model was run separately for deciduous, evergreen, and mixed forests. We also summed the forest cover fractions of all forest types to estimate the percentage of overall forest cover for each pixel.

The accuracy of the final forest cover map was assessed based on a stratified random sample using the mapped class proportions to define two strata: pixels with an overall forest proportion >0% (forest) and pixels with an overall forest proportion of 0% (non-forest). Within each stratum, we randomly selected 1000 MODIS pixel samples. Again, we used the Landsat maps to estimate the reference forest cover proportion for each pixel. We then assessed the agreement between the MODIS forest proportions and the Landsat-based proportions of deciduous, evergreen, and mixed forests by estimating the normalized root-mean-square error (NRMSE) between predictions and reference data:

1 $$NRMSE=\sqrt{\frac{\sum _{i=1}^{n}{({P}_{i}-{R}_{i})}^{2}}{n}}/\overline{R},$$

where *P*_*i*_ and *R*_*i*_ are the model prediction and reference for forest cover, respectively, for pixel *i* with a sample size of *n*, and $\overline{R}$ is the mean value of the reference.

### Comparison of tree cover maps in Central Asia

Two global tree cover products were compared with our MODIS forest cover map (CAFC) to determine the strengths and limits of all available maps for Central Asia. We selected the Global Forest Change (GFC) dataset and MODIS Vegetation Continuous Fields (VCF) for these comparisons. GFC is a state-of-the-art global forest change product by Hansen *et al*.^52^ with a 30-meter resolution. It estimates per-pixel tree cover percentage for 2000 and annual forest cover change between 2000 and 2013 using a rigorous data processing chain. To derive tree cover in 2010, we summarized forest changes during 2000 and 2010 and used the results to update the tree cover layer produced for the year 2000. Pixels labelled as forest loss during 2000–2010 were assigned 0% tree cover in 2010. Inversely, pixels mapped as forest gain before 2010 were labelled as 100% tree cover. In all, a number of pixels equal to 2.1% of the area with >25% tree cover in 2000 were relabelled. The MODIS VCF product was developed by the University of Maryland at a spatial resolution of 250 m^29^. The inputs for MODIS VCF include the 16-day surface reflectance composites of ten MODIS spectral bands. MODIS VCF Collection 5 (MOD44B) for the year 2010 was downloaded and used for the comparison. Several other global land cover maps provide categorical land cover information at a coarse resolution. However, we only selected continuous tree cover data because sub-pixel proportions are likely to capture heterogeneous and sparse forest cover better than discrete maps at that spatial resolution, and these data are more comparable to our map.

In the absence of information from GFC and MODIS VCF on specific forest types, we only selected the overall forest cover layer from the CAFC for the comparison. GFC was translated into a forest cover map for comparison with the CAFC. MODIS VCF was then compared with GFC without translation into forest cover because of the coarse resolution of MODIS VCF product. We followed this procedure to identify similarities and discrepancies between the forest cover products without losing detail. Comparisons were performed at the same spatial scale. First, all maps were re-projected to an Albers equal-area conic projection. Second, the GFC product was translated to a forest/non-forest map using a tree cover threshold of 30%. Third, we standardized all of the maps originating from the products to a resolution of 250-m by averaging all pixel values within a 250-m sampling unit. Because geolocation errors in remote sensing data and maps can influence per-pixel comparisons, we also included a comparison of larger pixel blocks by gradually decreasing the spatial resolution of the maps to 1 km and 5 km. Further, we only selected pixels with a tree cover or forest cover greater than 0% according to GFC, MODIS VCF, or CAFC. We also compared per-country forest cover statistics according to CAFC, GFC, MODIS VCF, and FAO FRA^68^. MODIS VCF was converted to a forest/non-forest map by applying a 30% tree-cover threshold before using it for per-country forest coverage calculations.

To improve information on worldwide forest cover, FRA 1990 and FRA 2000 included a remote sensing survey in tropical areas to complement national reporting. The FRA 2010 expanded the previous surveys to a global scale based on a systematic sampling approach with the aim of providing statistics on forest area change and land use dynamics at the regional, biome, and global scales^98^. We took forest data from the FRA 2010 and 2015 reports without adjustment since, according to the individual country reports, the national data had been adjusted and re-classified to comply with the FRA format, as well as because of a lack of details provided for the (re-) classification procedures^99^.

## Electronic supplementary material

Supplementary Information
