# Supplementary material for: Forest cover mapping in post-Soviet Central Asia using multi-resolution remote sensing imagery
Source: Sci Rep. 2017 May 2;7:1375. doi: 10.1038/s41598-017-01582-x (PMC5431049; doi:10.1038/s41598-017-01582-x)
Supplement: Supplementary file 1 — Supplementary Information [file 41598_2017_1582_MOESM1_ESM.doc]

Supplementary Information

**Forest cover mapping in post-Soviet Central Asia using multi-resolution remote sensing imagery**

He Yin, Asia Khamzina, Dirk Pflugmacher, Christopher Martius

A. Supplementary Figure Legends and Figures

B. Supplementary Tables

A. Supplementary Figure Legends and Figures


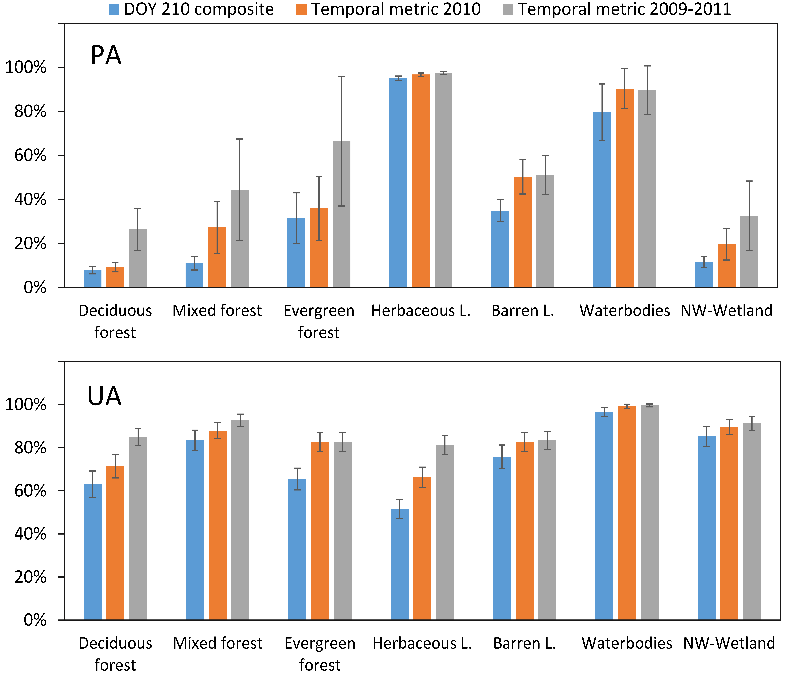


Figure S1 Producer’s accuracy (PA) and user’s accuracy (UA) with associated standard error using three sets of predictors.

**
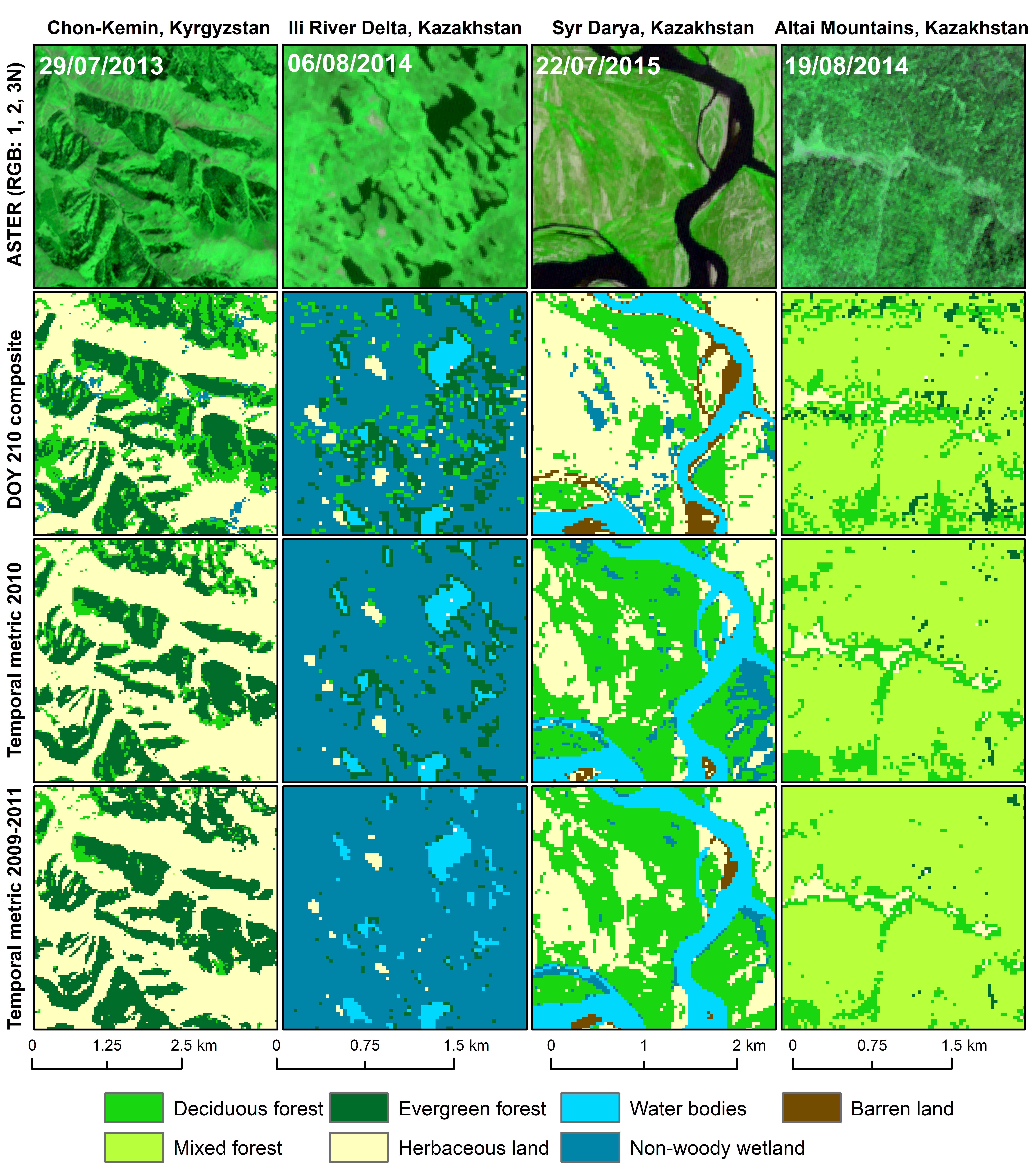
**

Figure S2 Spatial subsets of land cover classification from three datasets at four sites using 15-m resolution ASTER imagery for visual interpretation. ASTER images were provided by the U.S. Geological Survey: <https://earthexplorer.usgs.gov/metadata/9380/2169798387/>; <https://earthexplorer.usgs.gov/metadata/9380/2171323976/>; <https://earthexplorer.usgs.gov/metadata/9380/2177795558/>; <https://earthexplorer.usgs.gov/metadata/9380/2171382474/>.


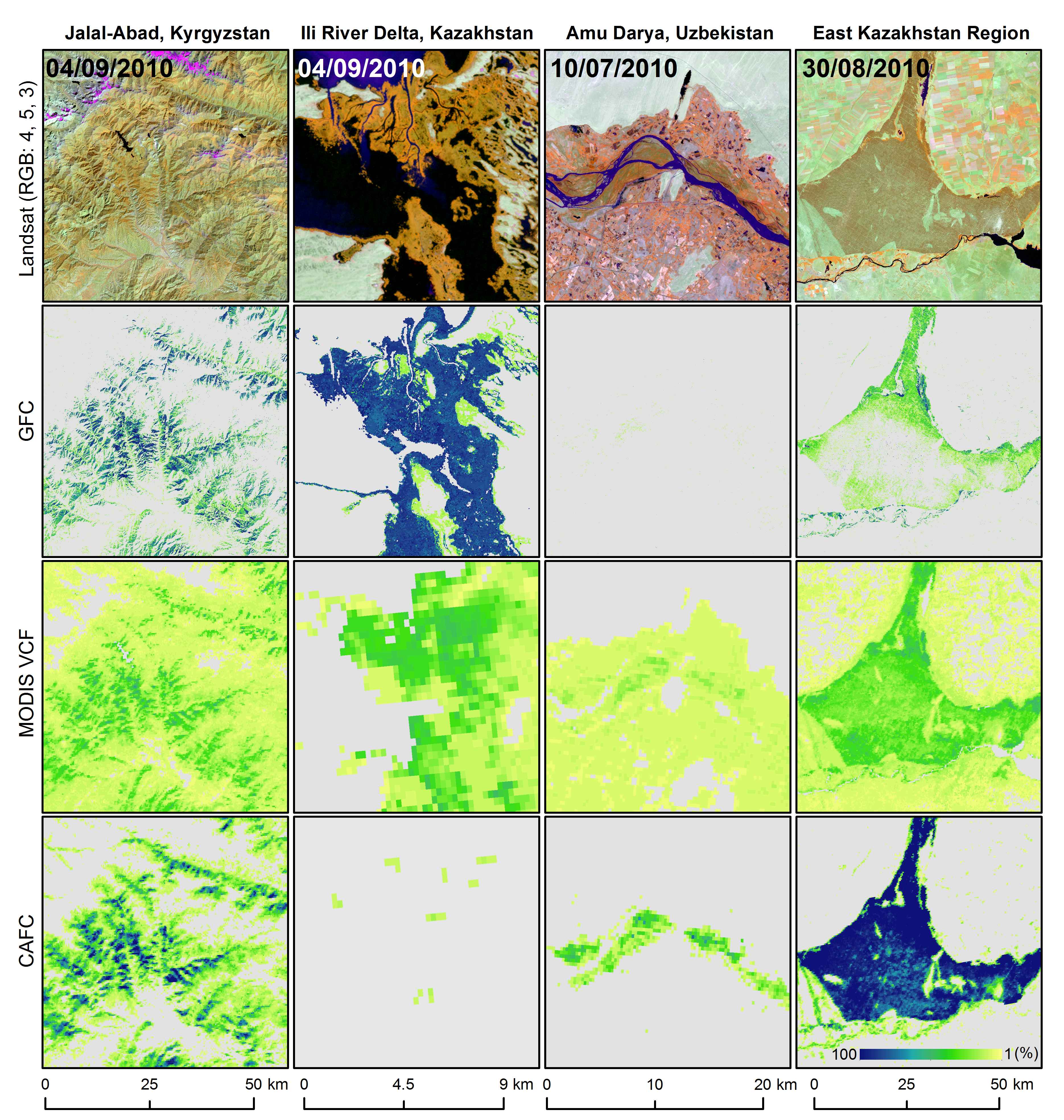


Figure S3 Spatial subsets of forest cover from CAFC (this study) and tree cover estimate from MODIS VCF and CATC at four sites using Landsat imagery for supporting visual interpretation. Landsat images were provided by the U.S. Geological Survey: <https://earthexplorer.usgs.gov/metadata/3119/LT51520312010247KHC00/>; <https://earthexplorer.usgs.gov/metadata/3119/LT51520292010247KHC01/>; <https://earthexplorer.usgs.gov/metadata/3119/LT51600312010191MOR02/>; <https://earthexplorer.usgs.gov/metadata/3119/LT51490252010242IKR01/>.


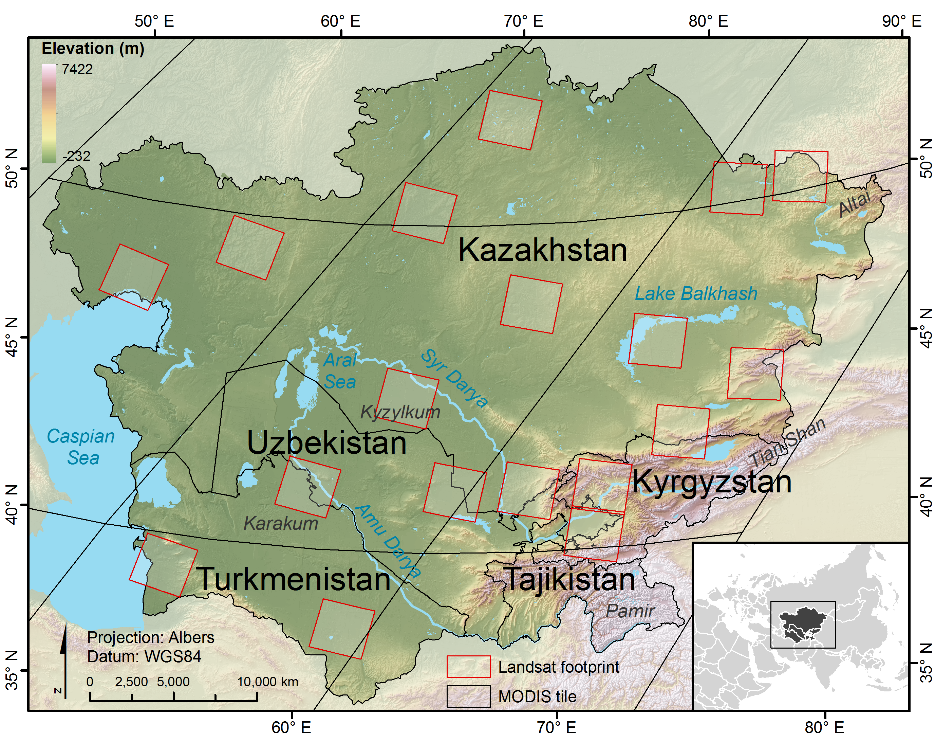


Figure S4 Location of the study area with the eighteen Landsat scenes used for land cover classification shown as red frames. This figure was produced using ArcGIS 10.3.

B. Supplementary Tables

Table S1 Error matrix with cells expressed as the estimated proportion of area (%) using three sets of predictors: DOY 210 composite, temporal metric 2010 and temporal metric 2009-2011. DF: deciduous forest, MF: mixed forest, EF: evergreen forest, HL: herbaceous land, BL: barren land, WD: water bodies, NW: non-woody wetland. UA: user’s accuracy, PA: producer’s accuracy. The UA and PA of the forest class in the aggregated forest/non-forest map is noted in the last row of each error matrix.

| DOY 210 composite | DF | MF | EF | HL | BL | WD | NW | ***UA*** |
| --- | --- | --- | --- | --- | --- | --- | --- | --- |
| DF | 1.09 | 0.24 | 0.06 | 0.24 | 0.01 | 0.01 | 0.07 | ***0.63*** |
| MF | 0.10 | 0.71 | 0.02 | 0.02 |  |  |  | ***0.83*** |
| EF | 0.08 | 0.21 | 0.97 | 0.10 | 0.00 | 0.03 | 0.09 | ***0.65*** |
| HL | 12.61 | 5.31 | 1.99 | 42.99 | 11.12 | 0.66 | 8.63 | ***0.52*** |
| BL | 0.03 |  |  | 1.84 | 5.98 | 0.03 |  | ***0.76*** |
| WD |  |  | 0.01 |  | 0.01 | 3.25 | 0.09 | ***0.97*** |
| NW | 0.04 |  | 0.03 | 0.04 |  | 0.09 | 1.17 | ***0.85*** |
| ***PA*** | ***0.08*** | ***0.11*** | ***0.32*** | ***0.95*** | ***0.35*** | ***0.80*** | ***0.12*** |  |
| Aggregated forest/ non-forest map: forest UA 0.86, PA 0.15 | | | | | | | | |

| Temporal metric 2010 | DF | MF | EF | HL | BL | WD | NW | ***UA*** |
| --- | --- | --- | --- | --- | --- | --- | --- | --- |
| DF | 1.24 | 0.18 | 0.04 | 0.22 |  | 0.01 | 0.05 | ***0.71*** |
| MF | 0.08 | 0.75 | 0.01 | 0.01 |  |  |  | ***0.88*** |
| EF | 0.02 | 0.10 | 1.13 | 0.08 |  | 0.03 |  | ***0.83*** |
| HL | 12.10 | 1.73 | 1.95 | 53.90 | 7.80 | 0.22 | 3.68 | ***0.66*** |
| BL |  |  |  | 1.49 | 7.89 | 0.13 | 0.03 | ***0.83*** |
| WD |  |  |  |  |  | 4.01 | 0.03 | ***0.99*** |
| NW | 0.03 |  | 0.01 | 0.03 |  | 0.04 | 0.92 | ***0.90*** |
| ***PA*** | ***0.09*** | ***0.27*** | ***0.36*** | ***0.97*** | ***0.50*** | ***0.90*** | ***0.20*** |  |
| Aggregated forest/ non-forest map: forest UA 0.90, PA 0.18 | | | | | | | | |

| Temporal metric 2009-2011 | DF | MF | EF | HL | BL | WD | NW | ***UA*** |
| --- | --- | --- | --- | --- | --- | --- | --- | --- |
| DF | 1.48 | 0.08 | 0.01 | 0.13 |  | 0.01 | 0.03 | ***0.85*** |
| MF | 0.03 | 0.79 | 0.01 | 0.02 |  |  |  | ***0.93*** |
| EF | 0.03 | 0.10 | 1.13 | 0.07 | 0.02 | 0.02 |  | ***0.83*** |
| HL | 4.07 | 0.81 | 0.54 | 66.24 | 7.60 | 0.27 | 1.90 | ***0.81*** |
| BL |  |  |  | 1.46 | 7.95 | 0.13 |  | ***0.83*** |
| WD |  |  |  |  |  | 4.02 | 0.01 | ***1.00*** |
| NW | 0.01 |  |  | 0.05 |  | 0.03 | 0.94 | ***0.91*** |
| ***PA*** | ***0.26*** | ***0.44*** | ***0.66*** | ***0.97*** | ***0.51*** | ***0.90*** | ***0.33*** |  |
| Aggregated forest/ non-forest map: forest UA 0.92, PA 0.40 | | | | | | | | |

Table S2 Number of Landsat images used for land cover classification

| Path/Row | Imagery number in 2009 | Imagery number in 2010 | Imagery number in 2011 | Total number | Average cloud cover (%) |
| --- | --- | --- | --- | --- | --- |
| 147/25 | 23 | 23 | 23 | 69 | 41.8 |
| 148/29 | 29 | 30 | 22 | 81 | 48.1 |
| 149/25 | 18 | 24 | 19 | 61 | 26.5 |
| 150/30 | 25 | 29 | 28 | 82 | 42.3 |
| 151/28 | 31 | 29 | 29 | 89 | 26.8 |
| 152/31 | 27 | 26 | 24 | 77 | 39.8 |
| 152/32 | 28 | 25 | 26 | 79 | 45.1 |
| 154/31 | 28 | 26 | 20 | 74 | 31.1 |
| 155/27 | 26 | 28 | 22 | 76 | 13.3 |
| 156/31 | 27 | 27 | 23 | 77 | 24.2 |
| 157/23 | 24 | 21 | 18 | 63 | 27.7 |
| 158/29 | 23 | 21 | 21 | 65 | 34.8 |
| 158/34 | 30 | 26 | 23 | 79 | 23.3 |
| 159/25 | 26 | 20 | 16 | 62 | 16.2 |
| 160/31 | 25 | 21 | 24 | 70 | 16.6 |
| 163/33 | 31 | 31 | 25 | 87 | 32.2 |
| 164/26 | 22 | 29 | 19 | 70 | 26.4 |
| 167/27 | 27 | 24 | 19 | 70 | 23.5 |

Table S3 Three sets of predictors generated from Landsat time series for land cover classification

|  | Single date composite (DOY 210) | Single year composite with temporal-metrics | Multiple year composite with temporal-metrics |
| --- | --- | --- | --- |
| Period | 2010 | 2010 | 2009-2011 |
| No. predictor | 6 spectral band composite | 30 spectral-temporal metrics | 30 spectral-temporal metrics |
